# A priori prediction of tumour response to neoadjuvant chemotherapy in breast cancer patients using quantitative CT and machine learning

**DOI:** 10.1038/s41598-020-67823-8

**Published:** 2020-07-02

**Authors:** Hadi Moghadas-Dastjerdi, Hira Rahman Sha-E-Tallat, Lakshmanan Sannachi, Ali Sadeghi-Naini, Gregory J. Czarnota

**Affiliations:** 1 Department of Medical Biophysics, University of Toronto, Toronto, ON Canada; 2 Physical Sciences Platform, Sunnybrook Research Institute, Sunnybrook Health Sciences Centre, Toronto, ON Canada; 3 Department of Radiation Oncology, Odette Cancer Centre, Sunnybrook Health Sciences Centre, Toronto, ON Canada; 4 Department of Radiation Oncology, University of Toronto, Toronto, ON Canada; 5 Faculty of Engineering, University of Waterloo, Waterloo, ON Canada; 6 Department of Electrical Engineering and Computer Science, Lassonde School of Engineering, York University, Toronto, ON Canada

**Keywords:** Breast cancer, Computer science, Breast cancer, Tumour biomarkers, Tumour heterogeneity, Biomedical engineering

## Abstract

Response to Neoadjuvant chemotherapy (NAC) has demonstrated a high correlation to survival in locally advanced breast cancer (LABC) patients. An early prediction of responsiveness to NAC could facilitate treatment adjustments on an individual patient basis that would be expected to improve treatment outcomes and patient survival. This study investigated, for the first time, the efficacy of quantitative computed tomography (qCT) parametric imaging to characterize intra-tumour heterogeneity and its application in predicting tumour response to NAC in LABC patients. Textural analyses were performed on CT images acquired from 72 patients before the start of chemotherapy to determine quantitative features of intra-tumour heterogeneity. The best feature subset for response prediction was selected through a sequential feature selection with bootstrap 0.632 + area under the receiver operating characteristic (ROC) curve (${\mathrm{A}\mathrm{U}\mathrm{C}}_{0.632+}$) as a performance criterion. Several classifiers were evaluated for response prediction using the selected feature subset. Amongst the applied classifiers an Adaboost decision tree provided the best results with cross-validated ${\mathrm{A}\mathrm{U}\mathrm{C}}_{0.632+}$, accuracy, sensitivity and specificity of 0.89, 84%, 80% and 88%, respectively. The promising results obtained in this study demonstrate the potential of the proposed biomarkers to be used as predictors of LABC tumour response to NAC prior to the start of treatment.

## Introduction

Breast cancer is the most commonly diagnosed cancer among women, accounting for about 30% of all new cases diagnosed each year in the United States and Canada^1,2^. Up to 20% of patients with breast cancer present with locally advanced breast cancer (LABC)^3–5^. LABC encompasses stage III and a subdivision of stage IIB disease, defined as tumours with a size of greater than 5 cm which may extend to the skin and/or chest wall. Current treatment for LABC includes neoadjuvant chemotherapy (NAC) followed by surgery and, if required, adjuvant radiation and/or hormonal therapy^6–8^. Despite multi-modal aggressive treatment, LABC patients still suffer from poor overall survival^4^. Whereas response to NAC has been demonstrated to correlate to patient survival, only about 20–30% of the patients realize a complete pathological response to standard NAC^5,9–12^. The standard approach to evaluate the tumour response to NAC is based on post-treatment anatomical imaging or histopathology on post-surgical specimens. However, the window to modify neoadjuvant treatment is closed at that time. Also changes in physical dimensions of tumour in response to chemotherapy can take several months to be detectable on anatomical imaging, and in some cases no change is evident in tumour size despite a pathological response to treatment. Predicting response to standard NAC before to early after the treatment initiation can facilitate treatment adjustments on an individual patient basis and potentially improve therapy outcome and patient survival.

A number of previous studies investigated different quantitative imaging techniques to evaluate chemotherapy response in breast cancer patients early after the start of treatment^13–22^. In^13^, dynamic contrast-enhanced magnetic resonance imaging (DCE-MRI) has been applied to predict the chemotherapy response in breast cancer. However, despite promising results, the cost and availability of MRI may restrict its utilization as a regular choice. Other researchers have used positron emission tomography (PET) to predict pathological complete response in women with breast cancer early after NAC^15,16^. The functional nature of measurements in PET also make it a potential choice to monitor tumour response, but it is expensive and requires using radionuclide contrast agents^17,18^. In recent years, diffuse optical imaging (DOI) has attracted the growing interest of researchers for monitoring and predicting breast cancer response to NAC^19,23^, since it can probe tissue oxygenation, and indirectly vascularity composition. In contrast to modalities such as CT and PET, DOI does not use ionizing radiation but it has not been adapted as an standard imaging modality in clinic and it requires long scan session to reconstruct volumetric images with reasonable resolution^17^. Ultrasound (US) is a non-ionizing and inexpensive modality that can probe the physical properties and specifically the microstructure of tissue without using any contrast agents, and hence it is a desirable choice for tumour response measurement to treatment^22,24–27^. Quantitative ultrasound (QUS) techniques have shown promising results in tissue characterization and tumour response monitoring^28,29^. However, in comparison with CT or MRI, the image quality is lower in US and usually it does not provide 3D images of the entire tumour volume in a facile manner.

Recent studies have also explored quantitative imaging techniques including DOI and QUS to predict LABC responses to chemotherapy prior to the start of treatment^23,30^. These studies hypothesized that the microstructure and metabolic characteristics of tumour can be linked to its aggressiveness and responsiveness to chemotherapy. They demonstrated that the optical and ultrasound features quantifying such characteristics can be predictive of tumour response to treatment. The micro-environment characteristics of tumour can also be quantified using quantitative computed tomography (qCT). CT Images are often acquired as part of the standard of care for LABC patients and, in conjunction with methods quantifying spatial heterogeneity, can potentially provide complementary information on tumour aggressiveness and responsiveness to treatment. Whereas the voxel size in CT images is frequently larger than cellular dimensions, changes in tissue microstructure could measurably alter the CT voxel intensities as a result of partial volume effects. As such, quantitative CT techniques have demonstrated promise in various tissue characterization applications including the measurement of bone mechanical properties^31^ and mineral density^32^, COPD diagnosis and staging^33,34^, the differentiation of primary lung cancer and granulomatous nodules^35^, pulmonary fibrosis assessment^36^, and the assessment of renal cell carcinoma differentiation^37,38^.

Although MRI is accurate in detecting primary inflammatory breast cancer lesions^39^, it may not always be accessible as the imaging modality of choice because of its cost and availability. As an alternative, CT images can provide diagnostic information about the spread of breast cancer and could be utilized for pre/post treatment examination^40^. Previous studies have demonstrated that contrast-enhanced CT (CE-CT) can considerably improve diagnostic information for breast cancer assessment compared to non-contrast CT^41,42^. The accuracy of CE-CT breast cancer diagnostic imaging for evaluation of both masses and calcifications has been demonstrated in multiple studies^43,44^. In^43^ CE-CT has been utilized to evaluate and monitor response to NAC in breast cancer patients during the course of treatment. Although breast imaging with CE-CT takes longer than CT, it is still much faster than MRI. Since CE-CT provides higher specificity over conventional radiography methods, it has been suggested that CE-CT should become the modality of choice for large population screening and extracting imaging biomarker in the emerging era of precision medicine^45^.

This study investigated, for the first time, the efficacy of qCT biomarkers to predict tumour response to NAC in LABC patients prior to the start of treatment. The qCT textural biomarkers were determined from pre-treatment CT images acquired from 72 LABC patients. The textural features were utilized in various types of machine learning algorithms to predict the tumour response to chemotherapy identified many months later based on standard clinical histopathology analysis of surgical specimens. The best qCT feature subset was selected through a multi-step feature ranking and feature selection process. The performance of the selected qCT biomarkers for response prediction were evaluated using different classifiers. Results indicated the qCT biomarkers in conjunction with an adaptive-boosting decision tree classifier predicted the pathological response of LABC tumours to chemotherapy with a cross-validated sensitivity and specificity of 80% and 88%, respectively. This study is a step forward towards the early prediction of cancer response to treatment using quantitative imaging biomarkers. Such prediction of therapy response could facilitate personalized medicine that is expected to improve survival and quality of life for cancer patients.

## Materials and methods

### Study protocol and data acquisition

This study was conducted under the regulations and guidelines in accordance with institutional research ethics board at Sunnybrook Health Sciences Centre (SHSC), Toronto, ON, Canada. All methods and experimental protocols were reviewed and approved by the research ethics board at SHSC prior to initiating the study. The study was open to all women aged 18 to 85 years who diagnosed with LABC and who planned to undergo a full course of NAC followed by surgery. In accordance with this, 72 eligible patients were enrolled in this study after receiving an informed consent. A core needle biopsy was performed for all patients to confirm the cancer diagnosis, and to determine the initial cellularity where possible, histological subtype, and the hormone receptor status of the tumour. Contrast-enhanced CT images of the breast were acquired for all patients at pre-treatment as part of the institutional standard of care. CT Scans were performed with a multi-slice CT scanner (LightSpeed, GE Medical Systems, Chicago, United States) using a helical acquisition mode. The scan parameters were X-ray tube current: 10–367 mA, tube voltage: 120 kV, slice size: 512 × 512 pixels, slice thickness: 2.5 mm, and pixel spacing: 0.8 × 0.8 mm. In order to measure the tumour size and assess the chest wall involvement, all patients underwent clinical MRI scans before and after the treatment as part of the institutional standard of care for LABC patients.

### Pathological evaluation of tumour response

All patients underwent breast surgery after the completion of their course of NAC. About two thirds of the patients underwent a mastectomy with the other patients went through a breast-conserving surgery (lumpectomy). The pathological response of tumour to NAC was assessed based on standard clinical histopathology on the surgical specimens. The specimens were stained with hematoxylin and eosin (H&E) and prepared on whole-mount 5″ × 7″ pathology slides which were digitized using a confocal scanner (TISSUEscopeTM, Huron Technologies, Waterloo, Canada). Patients were categorized into two groups of responders (R) and non-responders (NR) using a modified response (MR) grading system which was based on RECIST^46^ and histopathological criteria^47^ as before^30^. The MR score was defined as follows: MR 1: no reduction in tumour size; MR 2: up to 30% reduction in tumour size; MR 3: 30–90% reduction in tumour size or a very low residual tumour cellularity determined histopathologically; MR 4: more than 90% reduction in tumour; MR 5: no evident tumour and no malignant cells identifiable in sections from the site of the tumour; only vascular fibroelastotic stroma remains, often containing macrophages; nevertheless, ductal carcinoma in situ may be present. The histopathological analysis for each patient was performed by a board-certified pathologist who was kept blinded to the study results. The patients with a MR score of 1–2 (less than 30% reduction in tumour size) and 3–5 (more than 30% reduction in tumour size or with very low residual tumour cellularity) were determined as NR and R, respectively. In keeping with this, 56 and 16 patients were identified as responders and non-responders, respectively.

### Feature extraction and pre-processing

The regions of interest (ROI) were outlined manually on all slices of each 3D CT image to include the entire tumour within the breast region. Textural analysis was performed using a grey-level co-occurrence matrix (GLCM) method to determine first-order and second-order textural features from each ROI. The features parametric texture-maps were generated using texture analysis of grey-scale CT images and a sliding window approach using a 3 × 3 pixel window and a sliding step of 1 pixel. The GLCM was used to quantify statistically the angular relationship between neighbouring pixels with different intensities and the distance between them^48^. The CT numbers of all images were quantized to 128 Gy levels and the GLCMs were symmetrically computed over one pixel distance from each reference pixel at different directions, i.e. at angels of 0°, 45°, 90° and 135°. Eight textural features of entropy (ENT), contrast (CON), correlation (COR), maximum probability (MAX), mean (MEA), homogeneity (HOM), standard deviation (STD) and energy (ENE) were computed from each GLCM and then averaged over all four GLCMs. The textural features were extracted from all 2D slices covering the entire tumour volume. In order to account for the differences in the area of tumour cross-sections among different slices, a weighted averaging scheme, based on the ROI area, was used to compute the average feature values for each tumour. The median number of slices utilized to calculate the average features for each tumour was 10. In order to make the numeric range of the features consistent^49^, feature standardization was done using the robust scaler method. The scaler moves the median to zero and scales the data according to the range between the 1st and 3rd quartiles. The standardization was performed independently on each feature by computing the relevant statistics on the samples in the dataset. The inter-feature correlations were assessed using a Pearson correlation analysis to obtain the coefficient of determination (R^2^) for each feature pair.

### Feature selection and response prediction

Prior to the start of feature selection, features were ranked based on minimal-redundancy-maximal-relevance (mRMR) criterion^50^. A sequential forward feature selection (SFS) scheme was applied to find the best feature subset. The features were incrementally selected based on their mRMR ranking and the performance of the feature subsets was evaluated using the ${\mathrm{A}\mathrm{U}\mathrm{C}}_{0.632+}$^51–53^. The subset that yielded the highest ${\mathrm{A}\mathrm{U}\mathrm{C}}_{0.632+}$ was selected as the best feature subset at the end of the process (Supplementary Fig. 1). This subset was used to train the classifiers afterward.

In order to address the imbalance issue of the data, the minority group was oversampled to a double size using the SOMTE method^54^, while the majority group was undersampled by taking B = 200 bootstrapped samples with the same size of the oversampled minority group. The SMOTE method has shown promise for oversampling in different applications including medical data analysis^55–62^. The balanced training sets were prepared by combining the oversampled minority subset with each of the bootstrapped subsets from the majority group. These combined training sets were randomly shuffled to provide a proper mixture of samples from different classes. Before under/oversampling, one of the samples was randomly left out as the test sample for leave-one-patient-out (LOPO) cross-validation (n-fold; n = 72). Each of the training sets was utilized to train a classifier. Then, a majority vote over all B = 200 classifiers were used to predict the label of the test sample. This procedure was repeated until all the samples were tested (Supplementary Fig. 2). The training/testing procedures were performed using various types of classifiers including a support vector machine (SVM)^63^ (kernel = RBF, g = 1.85, c = 0.9), a decision tree (DT) classifier^64^ (maximum depth = 4, 100 estimators, learning rate = 0.1), a multilayer perceptron (MLP) neural network (NN)^65^ (hidden layer sizes = 9, optimizer = ADAM, alpha = 1e-4), a random forest (RF) classifier^66^ (maximum depth = 4, 100 estimators), adaptive boosting (AdaBoost)^67^ classifiers using SVM and DT as the weak learner and a hybrid classifier that consisted of SVM, RF and Adaboost-DT classifiers. The median of the three predictions of the hybrid classifier was used as its final prediction. All the classifiers were implemented in Python using Scikit-learn^68^ and a Dell PC OptiPlex 3,020 (Intel Core i5 3.30 GHz CPU, 8 GB RAM, Dell Inc, Round Rock, TX, U.S.A.) with a Windows 7 (64-bit) operating system (Microsoft, Redmond, WA, U.S.A.).

## Results

The clinical and pathological characteristics of participating patients are summarized in Table 1. The age of patients was in the range of 27–83 years with a mean and standard deviation of 52.7 ± 11.9 years. The primary tumour size was in the range of 1.3–12.8 cm with the mean and standard deviation of 5.5 ± 2.7 cm. The majority of the patients (93%) diagnosed with invasive ductal carcinoma (IDC), while 4% of the patients had invasive lobular carcinoma (ILC), and 3% had invasive metaplastic carcinoma (IMPC). Moreover, 62% and 60% of the patients had tumours with positive estrogen (ER +) and progesterone (PR +) receptors, respectively, whereas 30% of the patients had tumours with positive Her2/Neu receptor (HER2 +), and 25% of the patients had a triple negative tumour. For NAC, 50% of the patients received adriamyacin, cytotoxan followed by paclitaxel (AC-T), 42% received 5-fluorouracil, epirubicin, cyclophosphamide followed by docetaxel (FEC-T), 6% received doxorubicin, cyclophosphamide followed by docetaxel (AC-D) and 3% received doxorubicin and cyclophosphamide (TC). Also, all patients with HER2 + tumours received monoclonal antibody traztuzumab (TRA). The treatment regimen was not modified based on the imaging findings during this observational study.

**Table 1:** Clinical and pathological characteristics of patients.

**Characteristics**	**Mean ± Standard deviation/Percentage**
Age	52.7 ± 11.9 years
Initial tumour size	5.5 ± 2.7 cm
Histology	
	Invasive ductal carcinoma: 93.4%
	Invasive lobular carcinoma: 4.1%
	Invasive metaplastic carcinoma: 2.5%
Tumour grade	
	Grade I: 1.4%
	Grade II: 50.9%
	Grade III: 47.7%
Molecular features	
	ER + : 62.3%
	PR + : 59.7%
	HER2 + : 30.1%
	Triple negative: 25%
	ER + /PR + / HER2 + : 16.7%
	ER + /PR + /HER2−: 41.7%
	ER−/PR−/HER2 + : 8.3%
Residual tumour size	3.2 ± 4.3 cm
Response	
	Responding patients: 77.8%
	Non-responding patients: 22.2%

**Figure 1:**
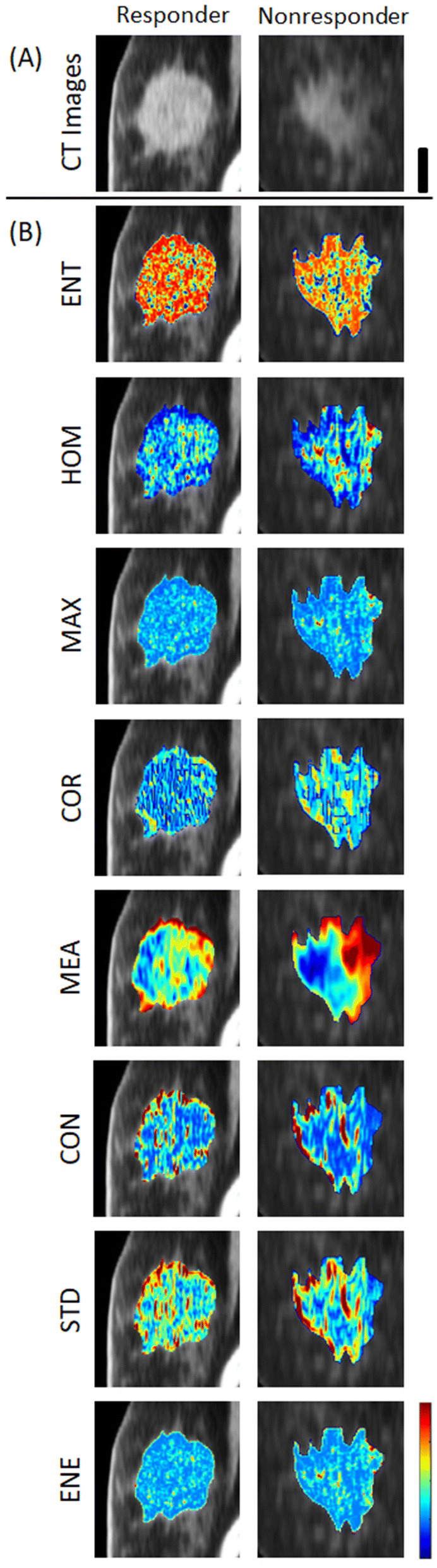
Representative CT images (**A**) with parametric map overlays (**B**) acquired for a responding and a non-responding patient. The parametric maps demonstrate entropy, homogeneity, maximum GLCM probability, correlation, GLCM mean, contrast, GLCM standard deviation and energy. The color bar represents a scale of the range [1.5, 2.5] for ENT, [0, 0.8] for HOM, [− 0.1, 0.8] for MAX, [− 0.2, 0.7] for COR, [0, 50] for MEA, [0, 100] for CON, [− 0.9, 8] for STD and [0.1, 0.8] for ENE. The scale bar represents 2 cm.

Figure 1 depicts representative CT images with parametric map overlays of the all 8 GLCM textural feature images for a representative responding and non-responding patients prior to the start of NAC. Different patterns of spatial variation were detectable in the corresponding parametric maps of the responding and non-responding patients. However, the differences were more evident in ENT, HOM, COR, MEA, CON and STD parametric maps. Additionally, the spatial patterns in HOM, STD and ENE parametric maps were generally comparable to those in CON, MAX and ENT, respectively.

**Figure 2:**
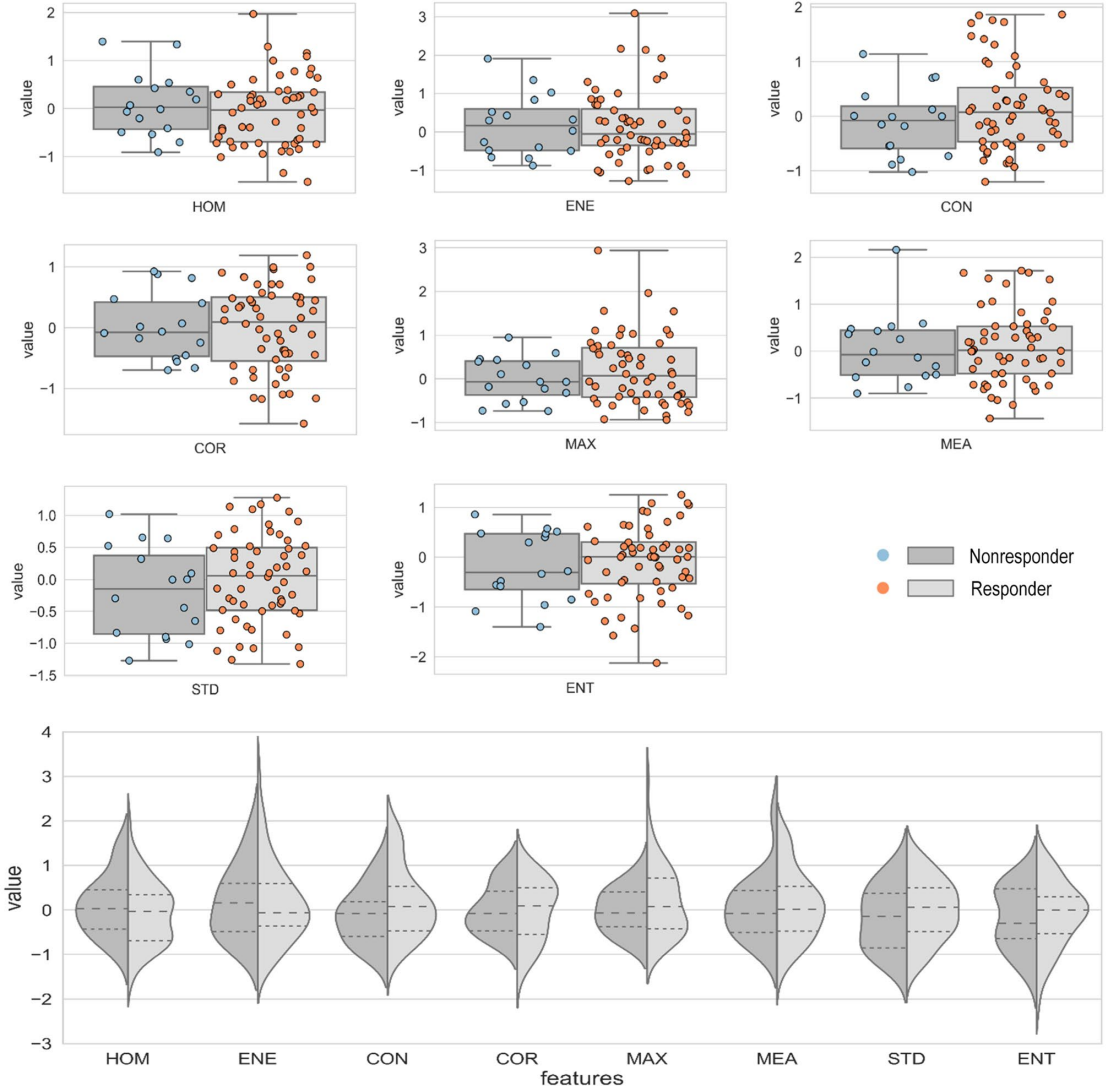
Statistical distribution of the feature values between the two groups of patients, i.e. responders (R) and non-responders (NR). The dash lines show the quartiles. All features were normalized according to a range between first and the third quartiles of their distribution.

The raw data and statistical distribution of the feature values between the responding and non-responding patients have been illustrated in Fig. 2. Although the range of feature values corresponding to the two patient cohorts demonstrated large overlaps, the mean values of ENE, COR, MEA, STD and ENT were considerably different. Similar to what observed in Fig. 1, ENE and CON as well as ENT and COR indicated comparable distributions.

**Figure 3:**
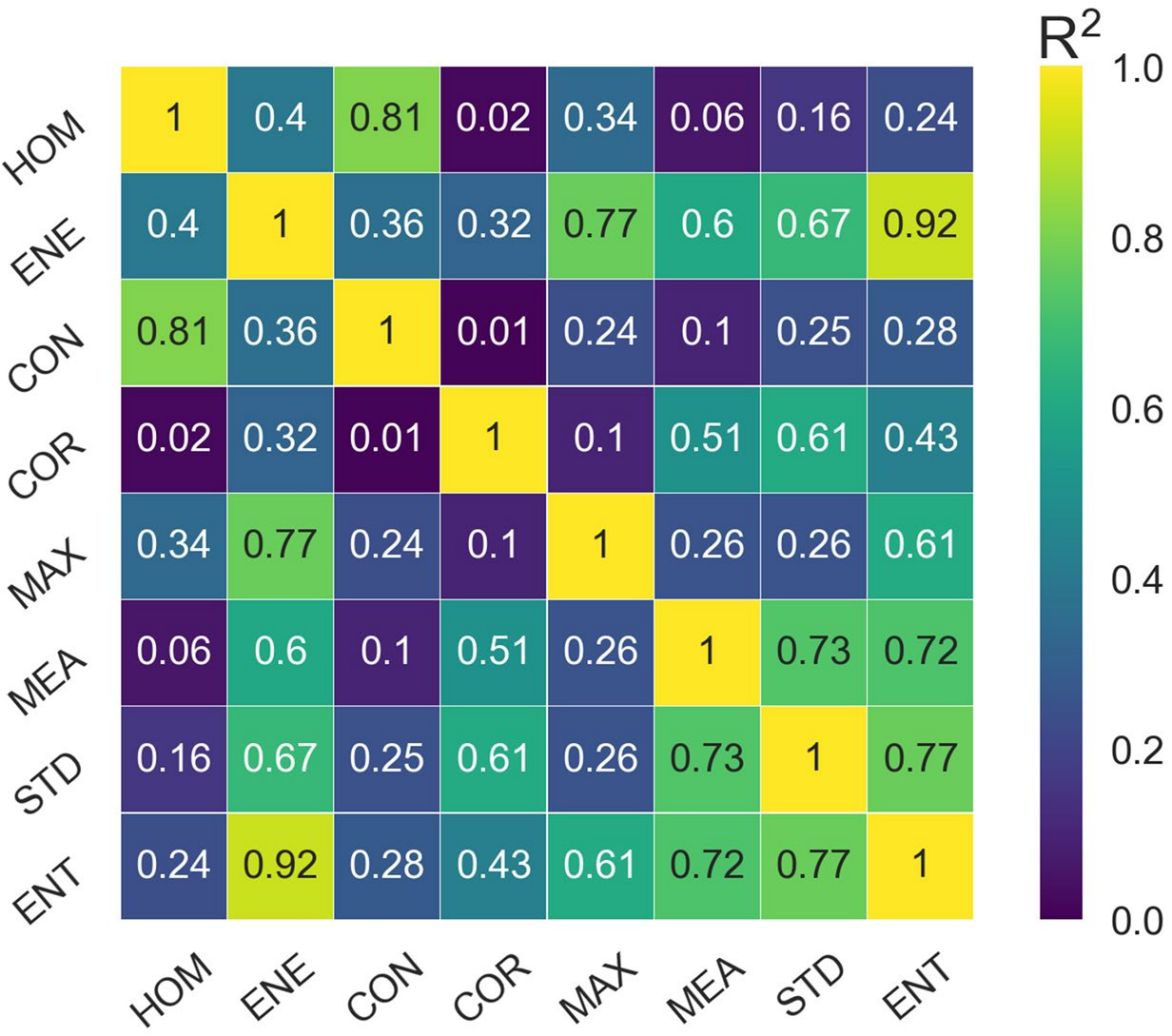
Heat map of the inter-feature correlations. The values show the coefficient of determination (R^2^).

Figure 3 demonstrates the inter-feature correlations. The highest coefficients of determination were found for ENE-ENT (R^2^ = 0.92), HOM-CON (R^2^ = 0.81), MAX-ENE (R^2^ = 0.77) and STD-ENT (R^2^ = 0.77). In contrast, CON-COR, HOM-COR, HOM-MEA and CON-MEA possessed the smallest coefficients of determination (R^2^: 0.01–0.1). The best feature subset obtained from feature selection included ENT, MAX, CON and MEA. This is in agreement with the results of inter-feature correlation analysis where HOM, STD and ENE parameters demonstrated high levels of correlation with CON, ENT and MAX (R^2^: 0.67–0.92), suggesting that these features potentially carry redundant information.

**Figure 4:**
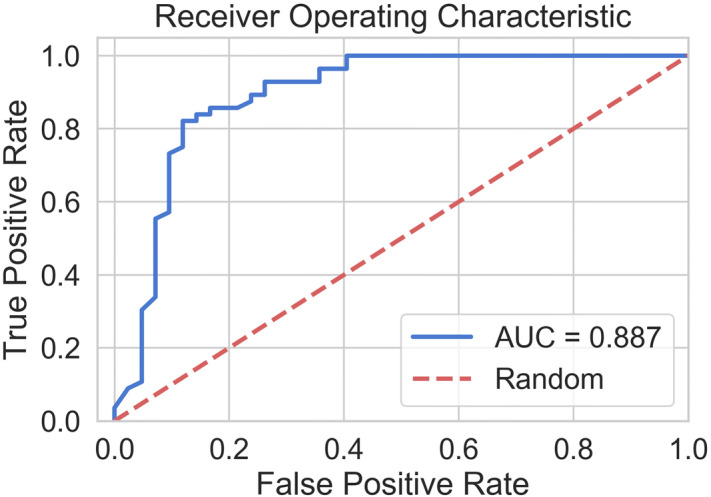
ROC curve of adaboost_DT classifier.

Table 2 presents the results of cross-validated response prediction using the selected feature subset with different classifiers. Results indicated that the Adaboost-DT methodology provided the most promising results among all the developed classifiers with cross-validated scores of ${\mathrm{A}\mathrm{U}\mathrm{C}}_{0.632+}$= 88.7%, accuracy = 83.7%, specificity = 88.1%, sensitivity = 80.4%, precision = 89.9% and f-score = 84.9%. Figure 4 shows the ROC curve of this classifier. The mean square error of prediction over all folds was 0.13 ± 0.26. The high value of standard variation was expected because there was only one prediction for each fold in the LOPO cross validation scheme. The SVM provided the fastest train and test performance on the LOPO bootstrapped samples and completed the classification process (Supplementary Fig. 2) quickly in 6.5 s. Among the applied classifiers, MLP was the slowest in classification and its performance was not outstanding. The hybrid method outperformed the SVM, but its result was not better than Adaboost-DT and was comparable to RF methodology.

**Table 2:** Summery of the classifier performance evaluation utilizing the best feature subset, i.e.[‘ENT’, ‘MAX’, ‘CON’, ‘MEA’]. The best result in each column is underlined.

**Classifier/score**	${{\varvec{A}}{\varvec{U}}{\varvec{C}}}_{0.632+}$** (%)**	**Accuracy (%)**	**AUC (%)**	**Specificity (%)**	**Sensitivity (%)**	**Precision (%)**	**F-Score (%)**	**Time (Sec)**
SVM	80.32	75.27	74.79	78.26	72.34	77.27	74.73	6.6
MLP	79.76	73.12	74.12	73.91	72.34	73.91	73.12	2,231
RF	84.15	80.65	80.23	84.78	76.60	83.72	80.00	139
Adaboost-SVM	83.11	78.49	78.03	82.16	74.47	81.40	77.78	1,493
Adaboost-DT	88.72	83.67	84.23	88.10	80.36	89.95	84.91	265
Hybrid	84.29	79.57	79.83	84.78	74.47	83.33	78.65	471

## Discussion and conclusions

This study, for the first time, explored the potential of utilizing qCT biomarkers to predict LABC responses to NAC prior to the start of treatment. Contrast-enhanced CT images were acquired from seventy two patients diagnosed with LABC before chemotherapy initiation. The patients were followed up during and after the course of chemotherapy and treatment outcomes were assessed using standard clinical methods. Several textural features were derived from each CT image using a GLCM approach and evaluated as potential chemotherapy response predictors. A sequential forward feature selection technique in conjunction with mRMR and ${\mathrm{A}\mathrm{U}\mathrm{C}}_{0.632+}$ methods were used to find the best feature subset. The best feature subset consisted of four features including ENT, MAX, CON and MEA. The best feature subsets were utilized in conjunction with different classifiers for response prediction. A LOPO cross validation was used to evaluate the performance of the developed classifiers for response prediction. The Adaboost-DT provided the best performance with an accuracy, f-score and ${\mathrm{A}\mathrm{U}\mathrm{C}}_{0.632+}$ of 83.7%, 84.91% and 88.7%, respectively. However, the SVM performed faster compared to the other classifiers.

The parametric maps generated for the GLCM textural features indicated considerable differences between the responding versus non-responding tumours prior to start of chemotherapy. The textural features quantify spatial variations in the CT voxel intensities that can characterize the underlying tissue micro-structure. The micro-structural characteristics of a tumour are potentially linked to its aggressiveness and responsiveness to chemotherapy, as demonstrated by a number of previous studies^30,69–71^. Although the spatial resolution of clinical CT images is low to visualize details of cellular structures, variations in tissue micro-structure can still be partially detected in these images as each voxel intensity maps the weighted average of attenuation coefficient corresponding to all elements within the voxel (partial volume effect)^33^.

The working hypothesis is that as tumours become more aggressive and less likely to respond to chemotherapy structurally tumour cells become more dysmorphic and as aggregates, they become more disorganized. The use of texture analysis on CT images becomes therefore a useful tool to characterize tumour micro-structure as it can be readily used to assess heterogeneity-linked changes. Analysis of the texture-based qCT parameters in conjunction with patient tumour responses to neoadjuvant chemotherapy can establish parameters that correlate with response to standard chemotherapy and can be thus used predictively.

A number of previous studies have investigated the applications of textural analysis on various imaging modalities including MRI, PET, DOI and US for breast cancer therapy response prediction^16,23,30,72–74^. Significant differences in texture parameters, including contrast, variance and entropy, has been reported in contrast-enhanced MR images between responding and non-responding patients prior to NAC treatment in^72,73^. Another study has demonstrated that the textural features of 18F-FDG PET images could be used to predict the pathological complete response to NAC after two cycles of treatment in both HER2- and HER2 + patients^16^. Additionally, it has been shown that DOI-based textural and mean-value parameters considerably change in LABC tumours in response to chemotherapy^19,23^. Tadayyon et al*.* have demonstrated that ultrasound textural features can quantify micro-structure and functional characteristics of tumour to predict the response to NAC^30^. On the other hand, recent studies have demonstrated that CT images can be utilized to simulate US images^46,74^, implying potential correlations between the anatomical and physiological information within US and CT. The results in the aforementioned studies are in agreement with observations in this study, in which microstructural characteristics of tumour were quantified by qCT parameters. Currently, contrast-enhanced CT imaging is used for detecting and characterizing breast tumours based on physical and anatomical measurements^46^. These available images can be used to extract the qCT biomarkers proposed in this study to predict the likelihood of standard chemotherapy response, potentially reducing the cost and side effects of futile treatments while improving the overall treatment outcome and patient survival.

In this study, a hybrid down-sampling/up-sampling method was used to balance the dataset. The SMOTE and bootstrapping methods were utilized for up-sampling the minority and down-sampling the majority, respectively. Results indicated that the misclassification rate of minority class remained the same after oversampling, e.g. 4 out of 16 versus 8 out of 32 for SVM classifier. In fact, the oversampling improved the results by preventing the classifiers from over-fitting toward the majority group.

The methodology proposed in this study for prediction of therapy response at pre-treatment can facilitate early therapy adjustments on an individual patient basis by modifying regimen, dose or priority of treatment options. The sensitivity (specificity) in this study refers to the proportion of the responders (non-responders) whose response to treatment was predicted correctly by the model. The specificity of the model could be shifted toward 100% by changing the threshold on the predicted probability of response. However, an optimal threshold should be determined very carefully as it makes a trade-off between the sensitivity and specificity of the model. Whereas predicting the non-responders to standard treatment reliably is crucial to implement early treatment adjustment for them, unnecessary modification of standard treatment for responding patients can affect their treatment outcome, increase their therapeutic side effects and influence their quality of life. Therefore, in this study the optimal thresholds were set such that an equal importance was assigned to both a high true positive rate as well as a low false positive rate.

In conclusion, this study demonstrated that qCT biomarkers can be used to predict LABC tumour response to chemotherapy at pre-treatment, with high sensitivity and specificity. The promising results obtained in this study is a step forward towards adapting quantitative imaging in conjunction with machine learning techniques for prediction of treatment response and outcome in cancer patients. Further investigations are, however, required to evaluate the efficacy of the proposed biomarkers on a larger cohort of patients. Nevertheless, given the ubiquitous use of CT scanning in medicine the markers are particularly interesting and the approach potentially applicable to a large number of different patients beyond the work presented here. Moreover, higher-order biomarkers of response may still remain unrevealed in breast CT images and have not been explored in this study due to the limited sample size. Therefore, more powerful feature extraction methods have been planned to be investigated in future studies on larger patient populations to extend the qCT biomarkers assessed in this study and evaluate them with higher statistical power.

## Supplementary information

Supplementary file1 (PDF 348 kb)
