# Supplementary material for: A priori prediction of tumour response to neoadjuvant chemotherapy in breast cancer patients using quantitative CT and machine learning
Source: Sci Rep. 2020 Jul 2;10:10936. doi: 10.1038/s41598-020-67823-8 (PMC7331583; doi:10.1038/s41598-020-67823-8)
Supplement: Supplementary file 1 — Supplementary file1 (PDF 348 kb) [file 41598_2020_67823_MOESM1_ESM.pdf]

Supplementary Information

A Priori Prediction of Tumour Response to Neoadjuvant Chemotherapy in Breast Cancer Patients using Quantitative CT and Machine Learning

Hadi Moghadas-Dastjerdi[1,2,3,4], Hira Rahman Sha-E-Tallat[2,5], Lakshmanan Sannachi[1,2,3,4], Ali Sadeghi-Naini[1,2,3, 6], Gregory J. Czarnota[1,2,3,4]

[1]Department of Medical Biophysics, University of Toronto, Toronto, ON, Canada.

[2]Physical Sciences Platform, Sunnybrook Research Institute, Sunnybrook Health Sciences Centre, Toronto, ON, Canada.

[3]Department of Radiation Oncology, Odette Cancer Centre, Sunnybrook Health Sciences Centre, Toronto, ON, Canada.

[4]Department of Radiation Oncology, University of Toronto, Toronto, ON, Canada.

[5]Faculty of Engineering, University of Waterloo, Waterloo, ON, Canada.

[6]Department of Electrical Engineering and Computer Science, Lassonde School of Engineering, York University, Toronto, ON, Canada.

Methods

Classifier Evaluation

The sequential forward feature selection scheme has been demonstrated in Supplementary Figure 1. In this flowchart, resubstitution method referred to a classifier that utilized all the samples to train and then to test. After sorting all the features based on mRMR, the best feature subset was selected using a forward sequential feature selection framework considering $AUC_{0.632+}$ as the criterion.

Supplementary Figure 2 illustrates the framework of the developed classification method which was consisted of two main blocks: pre-processing and classification. In the pre-processing block, one of the patients was left out as the test sample in each iteration. Then, B=200 bootstrapped samples were generated for the responders with the same size of oversampled subset for the non-responders. In the second block, the oversampled non-responders were combined with each of the 200 bootstrapped subsets for the responders to form 200 training sets. In next step, every training set were used to train a classifier. Then, these trained classifiers voted to predict the outcome for the test sample. This procedure was repeated to find the cross-validated prediction for all the patients through a leave-one-patient-out scheme.

The $AUC_{0.632+}$, accuracy, specificity, sensitivity, precision and f-score were obtained using the equations below:

$$Sensitivity = \frac{True\ Predicted\ Responders}{True\ Predicted\ Responders + False\ Predicted\ Nonresponders} \quad (1)$$

$$Specificity = \frac{True\ Predicted\ Nonresponders}{True\ Predicted\ Nonresponders + False\ Predicted\ Responders} \quad (2)$$

$$Accuracy = \frac{True\ Predicted\ Responders + True\ Predicted\ Nonresponders}{Totall\ Numbrer\ of\ patients} \quad (3)$$

$$Precision = \frac{True\ Predicted\ Responders}{True\ Predicted\ Responders + False\ Predicted\ Responders} \quad (4)$$

$$Recall = \frac{True\ Predicted\ Responders}{True\ Predicted\ Responders + False\ Predicted\ Nonresponders} \quad (5)$$

$$\text{F} - \text{Score} = \frac{2 \times Persicion \times Recall}{presicion + Recall} \tag{6}$$

$$AUC_{0.632+} = \alpha_b AUC'_b + (1 - \alpha_b) AUC \tag{7}$$

$$AUC'_b = max\{0.5, AUC_b\} \tag{8}$$

$$\alpha_b = \frac{0.632}{1 - 0.368\, R_b} \tag{9}$$

$$R_b = \begin{cases} 1 & if\ AUC_b \leq 0.5 \\ \dfrac{AUC - AUC_b}{AUC - 0.5} & if\ AUC > AUC_b > 0.5 \\ 0 & otherwise \end{cases} \tag{10}$$

where AUC_b is the AUC of the resubstitution method [1].

Reference

[1] B. Sahiner, H.-P. Chan, and L. Hadjiiski, "Classifier performance prediction for computer-aided diagnosis using a limited dataset," *Med. Phys.*, vol. 35, no. 4, pp. 1559–1570, Mar. 2008.

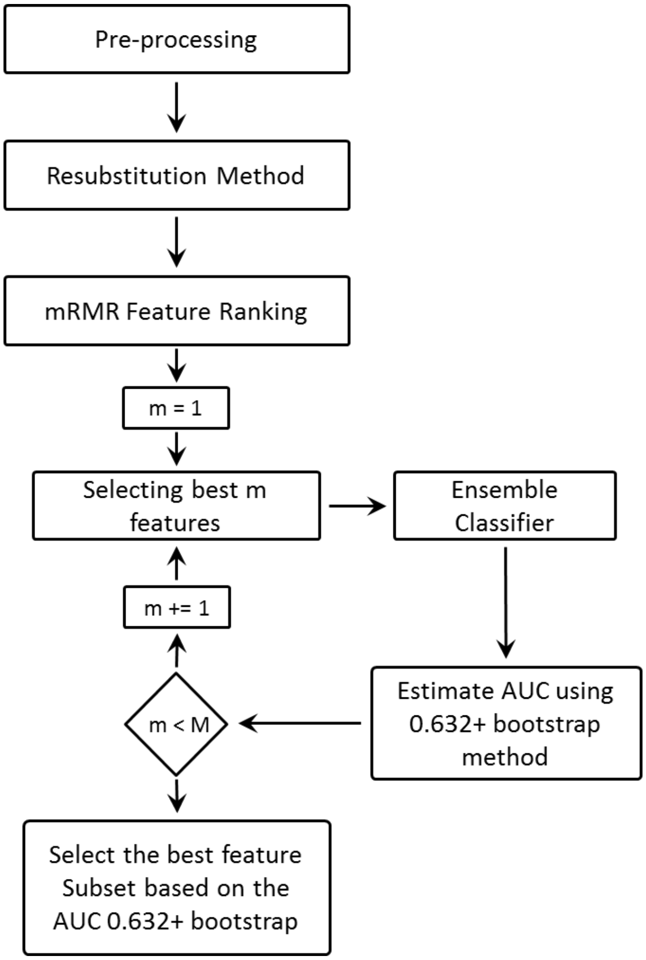

Supplementary Figure 1: The flowchart of the applied forward sequential feature selection algorithm. M is the total number of features.

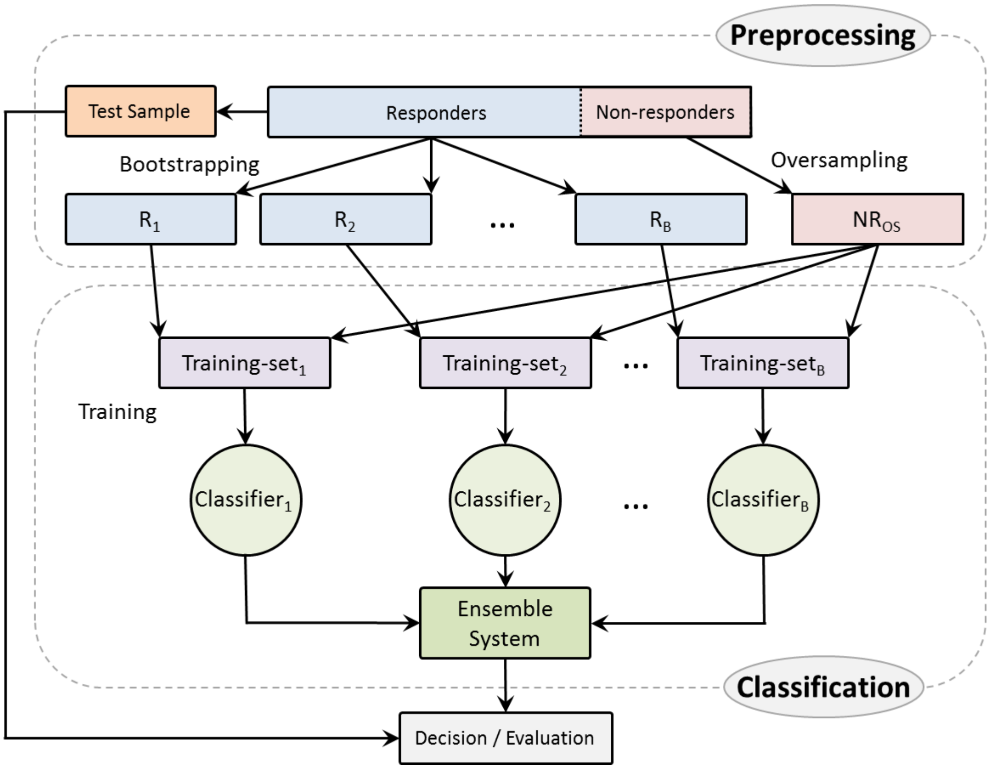

Supplementary Figure 2: The flowchart of the developed ensemble classifier. R_1 to R_B are the B=200 bootstrapped samples and NR_{OS} is the oversampled set of non-responders.
